# Genome sequences published outside of *Standards in Genomic Sciences,* January – June 2011

**DOI:** 10.1601/sigs.2044675

**Published:** 2011-06-30

**Authors:** Oranmiyan W. Nelson, George M. Garrity

**Affiliations:** 1 Editorial Office, Standards in Genomic Sciences and Department of Microbiology, Michigan State University, East Lansing, MI, USA

## Abstract

The purpose of this table is to provide the community with a citable record of publications of ongoing genome sequencing projects that have led to a publication in the scientific literature. While our goal is to make the list complete, there is no guarantee that we may have omitted one or more publications appearing in this time frame. Readers and authors who wish to have publications added to this subsequent versions of this list are invited to provide the bibliometric data for such references to the SIGS editorial office.

**Phylum *Crenarchaeota***
*“Metallosphaera cuprina”* Ar-4, sequence accession CP002656 [1]
*Thermoproteus uzoniensis* 768-20, sequence accession CP002590 [2]
*“Vulcanisaeta moutnovskia”* 768-28, sequence accession CP002529 [3]
**Phylum *Euryarchaeota***
*Methanosaeta concilii*, sequence accession CP002565 (chromosome), CP002566 (plasmid) [4]
*Pyrococcus* sp. NA2, sequence accession CP002670 [5]
*Thermococcus barophilus* MP, sequence accession CP002372 (chromosome) and CP002373 plasmid) [6]
**Phylum *Chloroflexi***
*Oscillochloris trichoides* DG-6, sequence accession ADVR00000000 [7]
**Phylum *Proteobacteria***
*Achromobacter xylosoxidans* A8, sequence accession CP002287 (chromosome), CP002288 (plasmid pA81), and CP002289 (plasmid pA82) [8]
*Acinetobacter baumannii* 3990, sequence accession AEOY00000000 [9]
*Acinetobacter baumannii* ST78, sequence accession AEPA00000000 [9]
*Acinetobacter baumannii*, sequence accession AEPA00000000 [9]
*Acinetobacter baumannii* MDR-TJ, sequence accession AEOE00000000 [10]
*Acinetobacter baumannii* TCDC-AB0715, sequence accession CP002522 (chromosome), CP002523 (p1ABTCDC0715) and CP002524 (p2ABTCDC0715) [11]
*Salmonella enterica* serovar Typhimurium UK-1 ATCC 68169, sequence accession CP002614 (chromosome), CP002615 (plasmid) [12]
*Acinetobacter calcoaceticus* PHEA-2, sequence accession CP002177 [13]
*Aeromonas caviae* Ae398, sequence accession CACP01000001 to CACP01000149 [14]
*Aeromonas veronii* B565, sequence accession CP002607 [15]
*Bordetella pertussis* CS, sequence accession CP002695 [16]
*Brucella melitensis* M111, sequence accession AFFB00000000 [17]
*Brucella melitensis* M28-12, sequence accession AFFA00000000 [17]
*Brucella melitensis* M5, sequence accession AFEZ00000000 [17]
*Brucella suis* S2, sequence accession AFFC00000000 [17]
*Brucella melitensis* M5-90, sequence accession CP001851 and CP001852 [18]
*Brucella melitensis* M28, sequence accession CP002459 and CP002460 [18]
*Burkholderia gladioli* BSR3, sequence accession CP002599 to CP002604 [19]
*Burkholderia phytofirmans*, sequence accession CP001052 to CP001054 [20]
*Burkholderia rhizoxinica* HKI 0454, sequence accession FR687359 (chromosome), FR687360 (pBRH01), FR687361 (pBRH02) [21]
*Campylobacter jejuni* S3, sequence accession CP001960 (chromosome) CP001961 (plasmid) [22]
Candidatus *Liberibacter solanacearum*, sequence accession CP002371 [23]
*“Citromicrobium* sp.” JLT1363, sequence accession AEUE01000000 [24]
*Cronobacter turicensis* LMG 23827, sequence accession NC_013282 to NC_013285 [25]
*Desulfovibrio africanus* Walvis Bay, sequence accession AFHE00000000 [26]
*Desulfovibrio desulfuricans* ND132, sequence accession AEUJ00000000 [27]
*Dickeya dadantii* 3937, sequence accession CP002038 [28]
*“Enterobacter mori”* LMG 25706, sequence accession AEXB01000000 [29]
*Erwinia amylovora*, sequence accession [30]
*Erwinia* sp. Ejp617, sequence accession CP002124 (chromosome), CP002125 (pJE01), CP002126 (pJE02), CP002127 (pJE03), CP002128 (pJE04), and CP002129 (pJE05) [31]
*Escherichia coli* AA86 KACC15541, sequence accession AFET00000000 [32]
*Escherichia coli* UM146, sequence accession CP002167 [33]
*“Gallibacterium anati”* UMN179, sequence accession CP002667, CP002668 [34]
*Gluconacetobacter* sp. SXCC-1, sequence accession AFCH0000000 [35]
*Haemophilus para*	 SH0165 serovar 5, sequence accession CP001321 [36]
*Herbaspirillum seropedicae* SmR1, sequence accession [37]
*Ketogulonicigenium vulgare* Y25, sequence accession CP002224 (chromosome), CP002225 (plasmid), and CP002226 (plasmid) [38]
*Methylocystis* sp. sp. Rockwell, sequence accession AEVM00000000 [39]
*Methylophaga thiooxydans* DSM010, sequence accession ABXT00000000 [40]
*Methylovorus* sp. MP688, sequence accession CP002258 [41]
*Neisseria gonorrhoeae* TCDC-NG08107, sequence accession CP002440 and CP002441 [42]
*Neisseria meningitidis* H44/76, sequence accession AEQZ00000000 [43]
*Neisseria meningitidis* WUE2594, sequence accession FR774048 [44]
*Oceanicaulis* sp. HTCC2633, sequence accession AAMQ00000000 [45]
*Paracoccus* sp. sp. TRP, sequence accession AEPN00000000 [46]
*Parvularcula bermudensis* HTCC2503^T^, sequence accession CP002156 [47]
*“Photobacterium mandapamensis”* svers. 1.1, sequence accession BACE01000001 to BACE01000031 [48]
*“Polymorphum gilvum”* SL003B-26A1T LMG 25793T, sequence accession CP002568, CP002569 [49]
*Pseudomonas savastanoi* pv. glycinea (Psg) B076, sequence accession AEGG01000000 [50]
*Pseudomonas savastanoi* pv. glycinea (Psg) race 4, sequence accession AEGH01000000 [50]
*Pseudomonas* sp. S9, sequence accession AFFX00000000 [51]
*Pseudomonas stutzeri* DSM4166, sequence accession CP002622 [52]
*Pusillimonas* sp. T7-7, sequence accession [53]
*Rhizobium etli* CNPAF512, sequence accession AEYZ00000000 [54]
*Rhodobacter sphaeroides* WS8N, sequence accession AFER00000000 [55]
*Roseobacter* sp. HTCC2038, sequence accession ABXE00000000 [56]
*Rubrivivax benzoatilyticus* JA2^T^, sequence accession AEWG00000000 [57]
*Ruegeria* TW15, sequence accession AEYW01000000 [58]
*Salmonella enterica* serovar Choleraesuis SCSA50, sequence accession CM001062-CM001063 [59]
*Salmonella enterica* serovar Dublin SD3246, sequence accession CM001151-CM001152 [59]
*Salmonella enterica* serovar Typhimurium 4/74, sequence accession CP002487 - CP002490 [59]
*Sphingomonas* sp. S17, sequence accession AFGG01000000 [60]
*Taylorella equigenitalis* MCE9, sequence accession CP002456 [61]
Unnamed strain IMCC1989, sequence accession AEVK00000000 [62]
Unnamed strain IMCC2047, sequence accession AEGL00000000 [63]
Unnamed strain IMCC3088, sequence accession AEIG00000000 [64]
Unnamed strain IMCC9063 SAR11 subgroup 3, sequence accession CP002511 [65]
*Variovorax paradoxus* S110, sequence accession [66]
*Vibrio anguillarum* pJM1, sequence accession AEZA00000000, AEZB00000000, AEZC00000000 [67]
*Vibrio furnissii* NCTC 11218, sequence accession CP002377 (chromosome I) and CP002378 (chromosome II) [68]
*Vibrio parahaemolyticus* clinical O4:K12 serotype, sequence accession AFBW00000000 [69]
*Vibrio rotiferianus* strain DAT722, sequence accession [70]
*Vibrio vulnificus* MO6-24/O, sequence accession CP002469 and CP002470 [71]
*Yersinia pestis* KIM D27, sequence accession ADDC00000000 [72]
*Yersinia enterocolitica* 3/O:9, sequence accession CP002246 (chromosome)_CP002247 (pYV plasmid) [73]
*Yersinia enterocolitica* subsp. *palearctica* 2 serogroup O:3, sequence accession FR729477 (chromosome) FR745874 (plasmid) [74]
**Phylum *Firmicutes***
*Bacillus amyloliquefaciens* LL3, sequence accession CP002634, CP002635 [75]
*Bacillus amyloliquefaciens* TA208, sequence accession CP002627 [76]
*Bacillus subtilis* BSn5, sequence accession CP002468 [77]
*Bacillus subtilis* subsp. *spizizenii* gtP20b, sequence accession AEHM00000000 [78]
*Bacillus thuringiensis* YBT-020, sequence accession CP002508 (chromosome), CP002509 (plasmid pBMB26), CP002510 (plasmid pBMB28 [79]
*“Bacillus thuringiensis* subsp. *chinensis”* CT-43, sequence accession CP001907.1, CP001910.1, CP001908.1, CP001915.1, CP001913.1, CP001911.1, CP001909.1, CP001917.1, CP001916.1, CP001914.1, CP001912.1 [80]
*Caloramator australicus* RC3T, sequence accession DRA000322 [81]
*Carnobacterium* sp. 17-4, sequence accession CP002563, CP002564 [82]
*Cellulosilyticum lentocellum* DSM 5427, sequence accession ADVF00000000 [83]
*Clostridium acetobutylicum* EA 2018, sequence accession NC_003030 [84]
*Clostridium botulinum* group III, sequence accession CP002410 - CP002415 [85]
*Clostridium botulinum* H04402 065, sequence accession FR773526 [86]
*Clostridium thermocellum* DSM1313, sequence accession CP002416 [87]
*Enterococcus faecalis* 62, sequence accession CP002491 - CP002495 [88]
*Erysipelothrix rhusiopathiae* ATCC 19414, sequence accession AP012027 [89]
*Eubacterium limosum* KIST612, sequence accession CP002273 [90]
*Exiguobacterium* sp. AT1b, sequence accession CP001615 [91]
*“Halanaerobium hydrogenoforman”*, sequence accession CP002304 [92]
*Lactobacillus amylovorus* GRL1118, sequence accession CP002338 [93]
*Lactobacillus animalis* KCTC 3501, sequence accession AEOF00000000 [94]
*Lactobacillus buchneri* NRRL B-30929, sequence accession CP002652, CP002653 (PLBU03), CP002654 (pLBU02), CP002655 (pLBU03) [95]
*Lactobacillus casei* (EP 164209630B1), sequence accession CP002616 and CP002617 [96]
*Lactobacillus casei* BD-II, sequence accession CP002618 and CP002619 [97]
*Lactobacillus coryniformis* subsp. *coryniformis* KCTC 3167, sequence accession AELK00000000 [98]
*Lactobacillus delbrueckii* subsp. *bulgaricus*, sequence accession CP000156 [99]
*Lactobacillus delbrueckii* subsp. *bulgaricus* ND02, sequence accession CP002341 and CP002342 [100]
*Lactobacillus farciminis* KCTC 3681, sequence accession AEOT00000000 [101]
*Lactobacillus helveticus* H10, sequence accession CP002429 (chromosome) and CP002430 (plasmid) [102]
*Lactobacillus plantarum* ST-III, sequence accession CP002222 [103]
*Lactobacillus reuteri* ATCC 53608, sequence accession CACS02000000 [104]
*Lactococcus garvieae* 21881, sequence accession AFCC01000000 [105]
*Lactococcus garvieae* UNIUD074, sequence accession AFHF01000000 [106]
*Lactococcus lactis* subsp. *lactis* CV56, sequence accession CP002365 through CP002370 [107]
*Leuconostoc fallax* KCTC 3537, sequence accession AEIZ00000000 [108]
*Leuconostoc gelidum* KCTC 3527, sequence accession AEMI00000000 [109]
*Leuconostoc inhae* KCTC 3774, sequence accession AEMJ00000000 [110]
*Listeria monocytogenes* J1-220, sequence accession AFBU00000000 [111]
*Listeria monocytogenes* J1816, sequence accession AFBU00000000 [111]
*Listeria monocytogenes* HCC23, sequence accession CP001175 [112]
*Melissococcus plutonius* ATCC 35311, sequence accession AP012200 (chromosome), AP012201 (plasmid) [113]
*Ornithinibacillus* TW25, sequence accession AEWH00000000 [114]
*Paenibacillus polymyxa* SC2, sequence accession CP002213 (chromosome) and CP002214 (plasmid) [115]
*Staphylococcus aureus* O11, sequence accession AEUQ01000000 [116]
*Staphylococcus aureus* straub O46, sequence accession AEUR01000000 [116]
*Staphylococcus aureus* T0131, ST239-MRSA-SCCmec type III, sequence accession CP002643 [117]
*Staphylococcus pseudintermedius* ED99, sequence accession CP002478 [118]
*Staphylococcus pseudintermedius* HKU10-03, sequence accession CP002439 [119]
*Streptococcus parauberis* KCTC11537BP, sequence accession CP002471 [120]
*Streptococcus suis* JS14, sequence accession CP002465 [121]
*Streptococcus thermophilus* ND03, sequence accession CP002340 [122]
*Turicibacter sanguinis* PC909, sequence accession ADMN00000000 [123]
*Weissella cibaria* KACC 11862, sequence accession AEKT01000000 [124]
**Phylum *Tenericutes***
*Mycoplasma alligatoris* A21JP2T, sequence accession NZ_ADNC01000000 [125]
*Mycoplasma crocodyli* MP145T, sequence accession CP001991 [125]
*Mycoplasma bovis* PG45 (ATCC 25523), sequence accession CP002188 [126]
*Mycoplasma haemofelis*, sequence accession FR773153 [127]
*Mycoplasma haemofelis* Ohio2, sequence accession AEVA00000000 [128]
*Mycoplasma suis* Illinois, sequence accession ADWK01000001 [128]
*Mycoplasma suis* KI3806, sequence accession FQ790233 [129]
**Phylum *Actinobacteria***
*Bifidobacterium bifidum* S17, sequence accession CP002220 [130]
*Bifidobacterium longum* subsp. *longum* BBMN68, sequence accession CP002286 [131]
*Corynebacterium pseudotuberculosis* I19, sequence accession CP002251 [132]
*Janibacter* sp. HTCC2649, sequence accession AAMN00000000 [133]
*Microbacterium testaceum* StLB037, sequence accession AP012052 [134]
*Mycobacterium bovis* BCG, sequence accession later [135]
*Nocardioides* sp. JS614, sequence accession CP000509, CP000508 [136]
*Saccharopolyspora spinosa* NRRL 18395, sequence accession AEYC00000000 [137]
*Streptomyces griseoaurantiacus*, sequence accession AEYX01000000 [138]
*Streptomyces griseus* XyelbKG-1, sequence accession ADFC00000000 [139]
*Streptomyces* sp. PP-C42, sequence accession AEWS01000000 [140]
*Verrucosispora maris* AB-18-032, sequence accession CP002638, CP002639 [141]
**Phylum *Chlamydiae***
*Chlamydia pecorum* E58, sequence accession CP002608 [142]
*Chlamydia psittaci* 6BC, sequence accession CP002586 (chromosome), CP002587 (plasmid) [143]
*Chlamydia psittaci* Cal10, sequence accession AEZD00000000 [143]
*Chlamydophila psittaci* RD1, sequence accession FQ482149 (chromosome) FQ482150 (plasmid) [144]
**Phylum *Spirochaetes***
*Borrelia burgdorferi*, sequence accession ABJZ02000001-5 (chromosome) CP001519 (Ip17) CP001518 (IP28-2) CP001523 (Ip28-4) CP001524 (Ip54) CP001522 (for cp26)_CP001517 (cp32-3) CP001520 (cp32-4) ABJZ02000006-7(Ip32-6) CP001521 (cp32-7)_CP001516 (cp32-12) [145]
*Treponema paraluiscuniculi* Cuniculi A, sequence accession CP002103 [146]
**Phylum *Fibrobacteres***
*Fibrobacter succinogenes* S85 S85, sequence accession CP001792 [147]
**Phylum *Bacteroidetes***
*Algoriphagus* sp. PR1, sequence accession AAXU01000000 [148]
*Bacteroides vulgatus* PC510, sequence accession ADKO01000000 [149]
*Kordia algicida* OT-1, sequence accession ABIB00000000 [150]
*Maribacter* sp. HTCC2170, sequence accession CP002157 [151]
*Riemerella anatipestifer* RA-GD, sequence accession CP002562 [152]
*Riemerella anatipestifer* RA-YM, sequence accession AENH00000000 [153]
**Phylum *Verrucomicrobia***
*Akkermansia muciniphila* ATCC BAA-835, sequence accession NC_010655 [154]
*Opitutus terrae* PB90-1, sequence accession CP001032 [155]
*“Chthoniobacter flavus”* Ellin428, sequence accession ABVL00000000 [156]
*“Pedosphaera parvula”* Ellin514, sequence accession ABOX00000000 [157]
**Phylum *Lentisphaerae***
*Victivallis vadensis* ATCC BAA-548, sequence accession ABDE02000001-ABDE02000027 [158]
